# Causal mechanisms of seismo-EM phenomena during the 1965–1967 Matsushiro earthquake swarm

**DOI:** 10.1038/srep44774

**Published:** 2017-03-21

**Authors:** Yuji Enomoto, Tsuneaki Yamabe, Nobuo Okumura

**Affiliations:** 1 Shinshu University, Ueda Campus, 3-15-1 Tokida, Ueda, Nagano, 386-8567, Japan; 2 Genesis Research Institute, Inc., 4-1-35 Noritake-shinmachi, Nishi-ku, Nagoya, Aichi, 451-0051, Japan

## Abstract

The 1965–1967 Matsushiro earthquake swarm in central Japan exhibited two unique characteristics. The first was a hydro-mechanical crust rupture resulting from degassing, volume expansion of CO_2_/water, and a crack opening within the critically stressed crust under a strike-slip stress. The other was, despite the lower total seismic energy, the occurrence of complexed seismo-electromagnetic (seismo-EM) phenomena of the geomagnetic intensity increase, unusual earthquake lights (EQLs) and atmospheric electric field (AEF) variations. Although the basic rupture process of this swarm of earthquakes is reasonably understood in terms of hydro-mechanical crust rupture, the associated seismo-EM processes remain largely unexplained. Here, we describe a series of seismo-EM mechanisms involved in the hydro-mechanical rupture process, as observed by coupling the electric interaction of rock rupture with CO_2_ gas and the dielectric-barrier discharge of the modelled fields in laboratory experiments. We found that CO_2_ gases passing through the newly created fracture surface of the rock were electrified to generate pressure-impressed current/electric dipoles, which could induce a magnetic field following Biot-Savart’s law, decrease the atmospheric electric field and generate dielectric-barrier discharge lightning affected by the coupling effect between the seismic and meteorological activities.

Since the seismic activities of the Matsushiro earthquake swarm occurred over a long period, i.e., 1965–1967, a considerable amount of reliable data has been reported on the seismic activities^1^,^2^,^3^,^4^,^5^,^6^ and the complexed seismo-EM activities^7^,^8^,^9^,^10^,^11^,^12^,^13^,^14^,^15^,^16^ as given in Figs 1,2 and 3, where the source of the map in Fig. 1 is Geospatial Information Authority of Japan website (http://maps.gsi.go.jp/#12/36.553224/138.221397/&base=std&ls=std%7C_ort&disp=11&lcd=_ort&vs=c1j0l0u0f0) created from the map data source: Landsat8 mosaic image (GSI, TSIC, GEO Grid/AIST), Landsat 8 image (courtesy of the U.S. Geological Survey), Geological Information Authority of Japan. The total number of felt earthquakes was ∼600,000, and the number of large earthquakes with magnitude *M* ≥ 4 was 88^1^,^2^. The highest magnitude reached *M* = 5.4. The focal depths ranged from 2–5 km^1^. The spatiotemporal variation in the seismic activities was divided into four stages^1^: The 1^st^–3^rd^ periods corresponded to three peaks of the felt earthquakes (c.f. Fig. 2a). As the epicentral region developed stage by stage in the northeast region, the vertical and horizontal deformation of the crust accelerated rapidly in the 3^rd^ stage (c.f. Fig. 2b)^1^. A fissure zone, 0.3 km wide and 1.8 km long, resulting from an underlying strike-slip fault, then developed near the northeast of Mt. Minakami where the open*-*surface cracks of a regular *en echelon* arrangement were successively constituted (c.f. Fig. 1 right-hand-insert)^3^,^4^,^5^. A large amount of ground water was expelled from the surface cracks accompanying the CO_2_ gas as shown in Fig. 2^7^. In the 4^th^ stage, the activities were diminished to the lower level^1^,^2^.

The rupture mechanism is reasonably understood as follows^18^,^19^,^20^,^21^,^22^. When the magma that caused the eruption (0.35 Ma) of Mt. Minakami, a Pleistocene andesite volcano, cooled, large amount of andesitic and CO_2_-rich water was dehydrated and became highly pressurized beneath the impermeable and seismic-wave reflective layer lying at ∼10 km deep^20^,^22^. The highly pressurized water then broke the impermeable layer and upwelled, passing through vertical flaws/cracks in the crust^18^,^19^,^21^, possibly caused by the shrinkage of the crust during the cooling process of the volcanic vent of Mt. Minakami. During upwelling of the water, the pressure decreased which resulted in degassing of CO_2_, volume-expansion and then the horizontal expansion of the opening of the vertical flaws/cracks (so called CO_2_/water eruption)^18^,^19^. The following supplementary information is added to the above-mentioned previous studies^18^,^19^,^20^,^21^,^22^: CO_2_ behaves as a supercritical fluid (sc-CO_2_) at a temperature *T* and pressure *P* above its critical point of *T*_c_ = 31 °C and *P*_c_ = 7.4 MPa, at which point sc-CO_2_ can dissolve various minerals in the crust causing corrosion cracking and local weakness in the surrounding crusts. *P*_c_ might be equivalent to the pressure at a depth of 3.3 km with a specific rock gravity *s*_g_ of 2.3, where the depth corresponded to that of the seismic zone. A phase change from sc-CO_2_ to gas occurred, leaving dissolved mineral deposits in the crack channels (referred to as sc-CO_2_ eruption). The sudden rock ruptures likely generated remarkable earthquake pop-like sounds as often noted during the active seismic periods^2^. Furthermore, as the critical point of water is *T* = 374 °C and *P* = 22.1 MPa, the dehydrated water/CO_2_ might have been in a supercritical state (sc-water/CO_2_) below the estimated depth of 12.4 km with an *s*_g_ of 2.3. Beneath the impermeable layer at ∼10 km deep the phase change of sc-water and the resulting volume expansion of the super-heated steam might have broken the impermeable layer.

The geomagnetic observation at the site C in Fig. 1 showed an anomalous increase in the total geomagnetic field on the order of 10 nT associated with the seismic activities in the 3^rd^ stage^13^ as shown in Fig. 3b. The geomagnetic variation exceeded a noise level of 0.2 nT, but could not be observed at the time of shallow the earthquakes of *M* ≥ 3.0^12^. The apparent absence of correlation between the earthquake occurrence and the geomagnetic variation indicated that the observed geomagnetic variation should not simply be attributed to the coseismic stress change.

The AEF observation from October to November 1966 demonstrated that the electric fields were often decreased by the felt-earthquakes^16^ (c.f. Fig. 3a). A similar decrease in the AEF may occur during an approach of a storm cloud with an electric dipole in the upper positive and lower negative range^23^. However, the AEF decreased with rapid alternative variation in the positive and negative directions on 22 Oct. 1966 (c.f. Fig. 3a), coinciding with favourable weather throughout the day as well as before and after. This conditions might indicate that electric dipoles generated in the underground seismic zone affected the ground electrification in a similar manner as the storm-cloud dipole. The AEF alternative variation became much more enhanced on the night of 14 Nov. 1966 (c.f. Fig. 3a). Although the weather recovered during the observed period, a cold front passed through during the day. The coupled interaction of seismic activities with meteorological variation, therefore, may have influenced the enhanced AEF variation.

The EQLs were photographed for the first time by T. Kuribayashi, an amateur photographer living in Matsushiro, as shown typical photos 1–3 in Fig. 1. Yasui^14^ evaluated the EQL reports as follows: Among 34 events recorded in the EQL candidates, 18 events could be identified; these events occurred in the period from the 1^st^ stage until the early 3^rd^ stage. As for the other 16 events, there were some fears of misjudgments due to other luminous phenomena, such as lightning or the sun or the moon, artificial lights and others (c.f. Fig. 3b). As seen in photos 1–3 in Fig. 1, the EQLs appeared in the mountainous areas within the seismic zone. Photo 3 was taken using a camera with a fish-eye lens on 26 September 1966, and it shows the scale of luminous zone, i.e., approximately 8 km (the yellow-coloured dotted circle in Fig. 1). The luminosity continued for 96 seconds. The felt earthquakes occurred at 03:11 and 03:37 L.T. but not at the time of the EQLs. The lack of direct coincidence between the EQLs and the earthquake occurrence indicated that the EQLs could not be explained by any model due to the coseismic stress change mechanism. Unlike the geomagnetic variation, which was notable in the 3^rd^ stage, the EQLs were commonly observed through the 1^st^–3^rd^ stages (c.f. Fig. 3b). This finding indicates that even if the source mechanism is identical, the way in which the source mechanism affects both seismo-EM phenomena differs. The EQLs are likely related to a certain mechanism involving the upwelling of CO_2_/water and the atmospheric condition, as the EQLs often occurred when a cold-front passed through^14^.

In terms of the stress-change mechanisms, the piezomagnetic model^24^ and the modified piezoelectric model^25^ have been proposed to explain either the geomagnetic variation or the EQLs. These models appear to provide a plausible explanation regarding whether the observed seismo-EMs were coseismic: however, this was not the case. A conceptual model was recently proposed that the electric fields at the ground-to-air interface due to an influx of stress activated charge carriers (positive holes) become so steep as to trigger corona discharges^26^,^27^. Corona discharges generate audible noise, but the Matsushiro EQLs did inaudible^14^.

An electrokinetic flow model^28^, where the electric current is generated due to groundwater flow passing through open cracks, seemed to be consistent with the observations in the 3^rd^ stage when the outflow rate of water at the fissure zone increased abruptly (c.f. the blue-coloured line in Fig. 2a). The estimated geomagnetic change of 4 nT^28^ was still lower than the observed change of 5–15 nT. The electrokinetic flow model would require rapid and implausibly continuous fluid flow^29^. This model, furthermore, could not explain why the EQLs occurred not only at the 3^rd^ stage, when the electrokinetic effect might be significant, but also at the 1^st^ and 2^nd^ stages when it might be less important.

In this context, a new source mechanism for the complexed seismo-EMs is needed to elucidate the underlying causal relationship between the hydro-mechanical and the electromagnetic processes. We consider that exoeletrons are emitted transiently from various trapped sites in lattice defects on fresh fractured surfaces^30^,^31^,^32^, and any gases that interacts with fractured fresh surfaces might be negatively electrified due to electron attachment reactions^32^. Taking into account the coupled electromagnetic interaction of the cracks with the gas flowing in as a working hypothesis for the seismo-EM phenomena^33^, we conducted laboratory experiments of uniaxial rock rupture coupled with high-pressure CO_2_ flow as described in the Methods: Fracture tests. The typical well-measured signals are shown in Fig. 4a for quartz diorite, the main rock constitution of the Matsushiro epicentre crust, with and without CO_2_ flow. The electric current was successfully measured for the rocks with CO_2_ flow, followed for approximately 2 msec after fully development of the crack. After the signal peak, the small alternating signals followed for ∼10 msec. The signal variations, observed both with and without CO_2_ flow, should be attributed to the vibrations excited by the electric field fluctuation between the charge-separated mating fracture surfaces. The observed peak currents *I*_(labo)_ are shown in Fig. 4b as a function of the gas-fracture interacting area *S*_(labo)_, defined as [thickness of the rock sample] × [width of collecting electrode]. The relationship between *I*_(labo)_ and *S*_(labo)_ can be expressed by


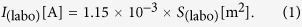


The present results suggest that as many water/CO_2_ gas-bearing pores are distributed in the shallow seismic zone, the dipole generation gave rise to short-term transient and temporally decaying electrification at the ground level. This process may provide a plausible explanation for the AEF variation (c.f. Fig. 3a) during the active earthquake periods.

During the 3^rd^ stage, as the geomagnetic intensity level gradually increased^13^, the successive opening of *en echelon*-type cracks, resulting from an underlying strike-slip fault, was observed in the fissure zone^3^,^4^,^5^. The laboratory experiments suggest that whenever an *en echelon* crack array extended from the deep seismic zone formed as a result of coupled left-lateral movement interactions and sc-CO_2_ eruptions, large vertical dipole currents *I*_(field)_ were generated with induced observable geomagnetic variation following Biot-Savart’s law. Based on the model shown in Fig. 4(c), we then estimated the geomagnetic variation |Δ***B***| assuming a line dipole current *I*_(field)_ element as follows.


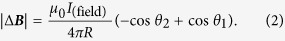


where *μ*_0_ is the permittivity of free space *μ*_0_ = 4π × 10^−7^ WbA^−1^m^−1^, *θ*_1_**and *θ*_2_ denote the angle shown in Fig. 4c, and *R* is the distance between the observation site and the fissure zone. We assumed that Eq. (1) holds even during the field-level rupture processes. The degree of geomagnetic variation |Δ***B***| is shown in Fig. 4d as a function of *R* for dipole lengths of 1.5 km and 3 km, where the dipole currents were estimated to be 450 A and 900 A using Eq. (1) for rupture areas of 0.3 km × 1.5 km = 0.45 km^2^ and 0.3 km × 3 km = 0.9 km^2^, respectively. These rupture areas are comparable to those associated with earthquakes of *M* = 3.6 and 3.9, respectively^34^. The estimated |Δ***B***| values are in reasonable agreement with those of the observed total geomagnetic variation level^13^. The present results suggest that because the fissure zone with *en echelon* crack arrays extended 1 km to 2.3 km from the geomagnetic observation site C in Fig. 1, it was located within a sensitive region that was not too distant or close for the detection of seismo-geomagnetic variation.

Assuming the rapid alternative AEF variations, such as those observed on 14 Nov. 1966 (c.f. Fig. 3a), which might be the source mechanism for the EQLs, we conducted the discharge experiments of the model fields as described in the Methods: Discharge tests. Figure 5a and b shows the model field of the embankments with and without twigs of an evergreen needle-shaped tree at an AC 15 kHz, respectively. The case in which twigs were included showed pale bluish-purple-coloured lights, similar to those of the eye-witnessed EQLs, but less overall lights than the case without the twigs. The twigs clearly act as a dielectric material for dielectric barrier discharge (DBD); so-called silent (inaudible) discharge, with lower discharge current than the spark discharge (SD) with audible noises, as seen in Fig. 6. The spectrum lines of the lights mainly consist of 2^nd^ positive nitrogen molecules within the wavelength range of 380–440 nm (c.f. Fig. 5c). The DBD due to AEF variations resulting from the coupled interaction of thunderstorm and seismic activities thus might be responsible for the EQLs, where trees covering the mountainous area played an important role in the silent EQLs.

In summary, a set of hydro-mechanical processes and the associated seismo-EMs are consistently explained with the present model as illustrated in Fig. 7, which improved the previous studies^20^,^22^. This is the first reported comprehensive modelling study to describe the causal relationships among a diverse set of seismic anomalies associated with the 1965–1967 Matsushiro earthquake swarm.

## Methods

### Fracture tests

**Figure 8:**
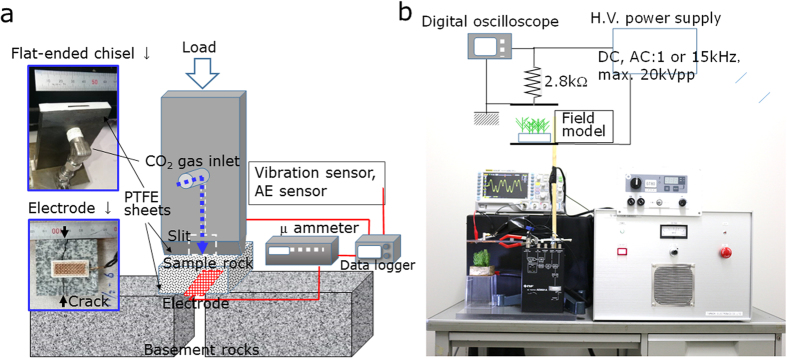
Experimental set up **(a)** Universal tester for measurement of electrified gas flow associated with rock fracture: the upper inset shows the PTFE sealed slit at the flat ended chisel and the lower insert shows a copper-mesh electrode equipped on the back side of the sample rock. **(b)** The block diagram and the view of the discharge experiment set up of the model field with or without twigs of an evergreen needle-shaped tree (*Monterey cypress Goldcrest*) using a high-voltage power supply of max.20 kV DC or AC 1 kHz and 15 kHz.

The experimental set up involving to a universal testing machine is shown in Fig. 8a. A flat-ended chisel, made of hardened carbon steel S45C with Brinell hardness of ∼230, was loaded against an as-received rock block (normally 50 × 50 × 20 mm in size); quartz diorite, crustal rock of the Matsushiro epicentral zone. CO_2_ gas at the pressure ranging from 0.3 to 0.5 MPa and at room temperature was introduced into a flow-channel inside the chisel. At an instant when the rock was subjected to guillotine-type fracture at a load of ∼12 kN, the pressurized CO_2_ gas flowed into the crack gap of the rock passing through the open slit (1 mm × 20 mm) equipped with the flat-ended chisel. To this end, a seal made of a 1-mm-thick PTFE sheet was set between the chisel of the flat-ended area around the open slit and the rock surface (c.f. Fig. 8a: upper insert). Undesirable three-point-type fractures of the rock were often observed, where the crack initiates from the bottom side to the top. Therefore, to ensure the crack could initiate from the upper side to allow CO_2_ gas to flow, the four corners of the upper rock surface were suppressed loosely with clamps (not shown in Fig. 8a). A clean copper mesh #20 electrode was set on the bottom of the rock (see the lower insert of Fig. 8a and biased at +77 dc volts to collect negatively charged gas flow. Two-ply sheets consisting of PTFE, 1 mm thick each, were set between the sample rock and the basement rocks of fine-grained granite in order to assist the horizontal displacement when the crack was opened. After the tests, work clay was pressed on the cracked rock surface to measure the gap size which typically ranged from 0.4 mm to 1 mm.

### Discharge tests

An experimental set up for discharge tests in an open-air room is shown in Fig. 8b, where a model field of embankment filled with wet soil (the resistivity was ∼260 ohm. m) with or without twigs of an evergreen needle-shaped tree (*Monterey cypress Goldcrest*) was set between the electrodes with separation distance of 10 cm and subjected to high voltage up to 20 kV DC or AC 1 kHz and 15 kHz. Discharge lights were photographed using a CMOS camera with ISO sensitivity of 25,600 at an exposure time of 5 sec. The spectrum was analyzed using a high-sensitivity spectral radiance meter. The discharge currents were measured in terms of voltage drop across 2.8 k resistor as shown in the block diagram of Fig. 8b^9^.

## Additional Information

**How to cite this article:** Enomoto, Y. *et al*. Causal mechanisms of seismo-EM phenomena during the 1965–1967 Matsushiro earthquake swarm. *Sci. Rep.* **7**, 44774; doi: 10.1038/srep44774 (2017).

**Publisher's note:** Springer Nature remains neutral with regard to jurisdictional claims in published maps and institutional affiliations.

## Supplementary Material

Supplementary Information

## Figures and Tables

**Figure 1:**
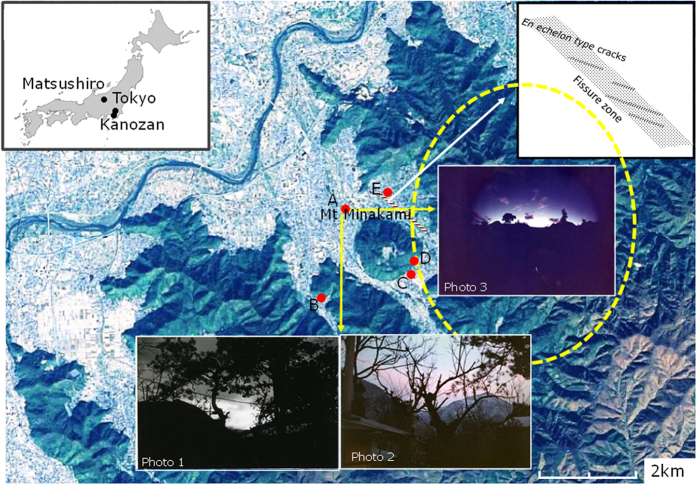
Locations of typical observation sites in Matsushiro area map. Source: Geospatial Information Authority of Japan website (http://maps.gsi.go.jp/#12/36.553224/138.221397/&base=std&ls=std%7C_ort&disp=11&lcd=_ort&vs=c1j0l0u0f0) created from the map data source: Landsat8 mosaic image (GSI, TSIC, GEO Grid/AIST), Landsat 8 image (courtesy of the U.S. Geological Survey), Geological Information Authority of Japan. Photos 1–3: photographed by T. Kuribayashi, Collections of Matsushiro Earthquake Center^14^. (**A**) The EQLs observation site: (**B**) the AEF observation site (Matsushiro Seismological Observatory), JMA^16^: (**C**) the geomagnetic observation station by ERI, University of Tokyo^3^,^4^,^5^,^6^,^7^: (**D**) the levelling resurvey point where the maximum upheaval movement was observed by ERI^6^: (**E**) the water outflow observation sites at the Kasuga hot spring^17^. Yellow circle: the EQL area estimated from Photo 3 with a fisheye lens. The insert on the right-hand side illustrates the fissure zone with *en echelon* crack arrays^3^,^4^,^5^.

**Figure 2:**
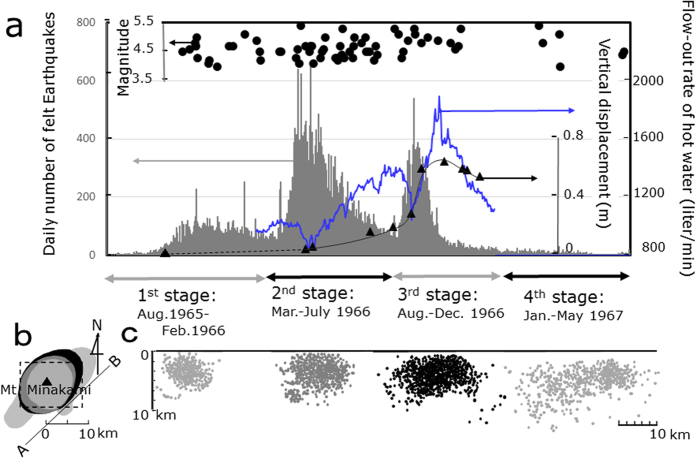
Spatiotemporal variation of seismic activities in the 1965–1967 Matushiro earthquake swarm. (**a**) Temporal variation of the daily number of felt earthquakes (grey bar)^2^, earthquakes of *M* ≥ 4(•)^2^, the outflow rate of spring water (blue line) at site E^17^ and the change in heights at site D in Fig. 1 (▴)^6^; **(b)** spatial distributions of the Matsushiro earthquake swarm in the four stages^1^ where the dotted square corresponds to the area of Fig. 1; (**c**) spatiotemporal variation of the focal distributions projected onto the vertical plane in the (A,B) direction of the four respective stages^1^.

**Figure 3:**
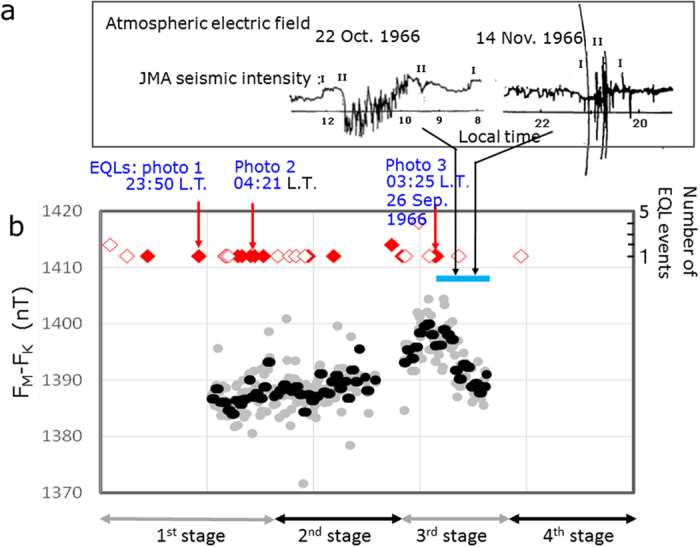
Temporal variation of seismo-EM phenomena. **(a)** Two typical AEF signals showing rapid alternative variations in the positive and negative directions for earthquakes I and II at the JMA seismic intensity^16^, **(b)** (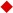) and (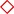): number of observed EQLs with the level of confidence of the seismic origin and without the level, respectively^14^. (•) and (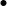): five-days and one-day mean temporal distribution of the total geomagnetic intensity at the Matsushiro site C in Fig. 1 with respect to the Kanozan site shown in the left insert of Fig. 1, respectively^7^,^8^,^9^,^10^,^11^,^12^,^13^. Sky-blue bar: the period of the AEF observation at site B in Fig. 1^16^.

**Figure 4:**
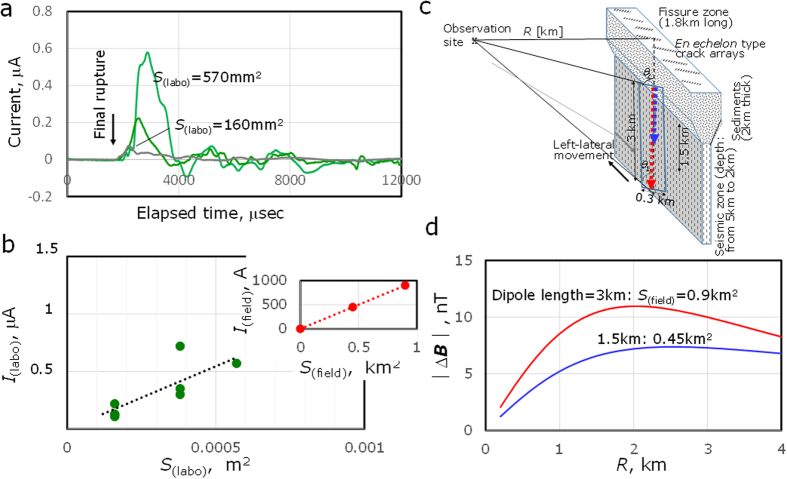
Electric currents due to the fracturing rocks (left) and the induced geomagnetic effects (right). **(a)** Current due to the fracturing of quartz diorite with and without CO_2_ gas flow (green and grey, respectively), **(b)** *I*_(labo)_ vs *S*_(labo)_. The dotted line is the approximated relationship of Eq. (1). Insert in the upper right: *I*_(field)_ vs *S*_(field)_ of 0.45 and 0.9 km^2^ in the fault zone as estimated using Eq. (1) and (**c**) the fault model showing a dipole current generated by the coupling interaction between CO_2_ gases and fracutring rocks. The red and blue arrows indicate a dipole current of the lengths, 3 km and 1.5 km, respectively. **(d)** |Δ***B***| estimated from Eq. (2) vs *R*.

**Figure 5:**
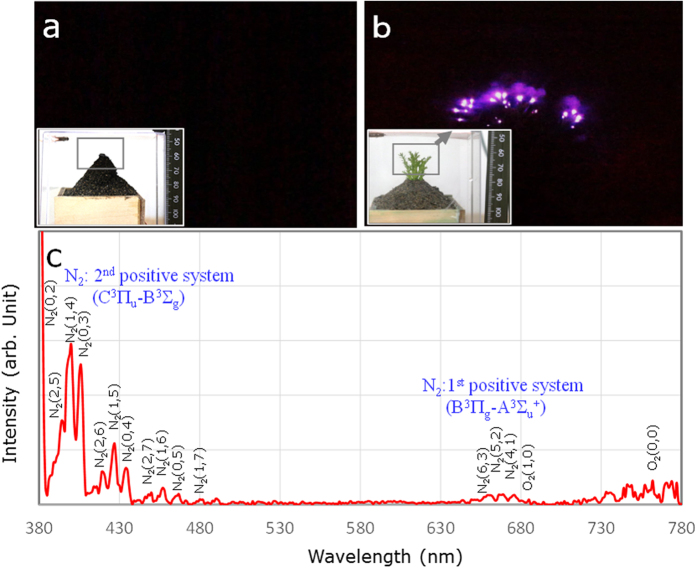
Laboratory discharge experimental results using the model-field. **(a)** Wet embankment without twigs of evergreen needle-shaped tree and **(b)** with the twigs at a 20 kV AC 15 kHz: **(c)** spectrum of the discharge lights of (**b**).

**Figure 6:**
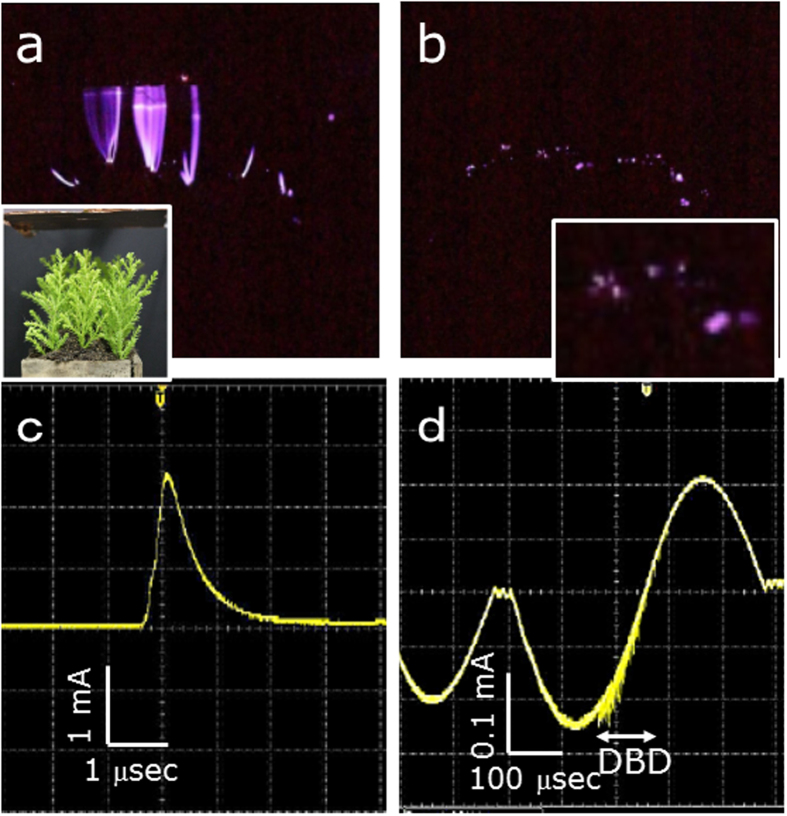
Laboratory discharge experimental results using the model-field (continued). **(a)** and **(b)** show the discharge lights of a model field shown in the insert of (a) at a 20 kV DC and AC 1 kHz, respectively. The insert of (b) show the DBD lights enlarged. The exposure time was 5 sec. **(c)** and **(d)** are their respective current signals. Note that the current for DBD was as small as one-tenth of the spark discharge SD. The DBD lights at AC 1 kHz in (b) become darker than that at 15 kHz (c.f. Fig. 5b) because the number of DBDs is reduced at the same exposure time of 5 sec.

**Figure 7:**
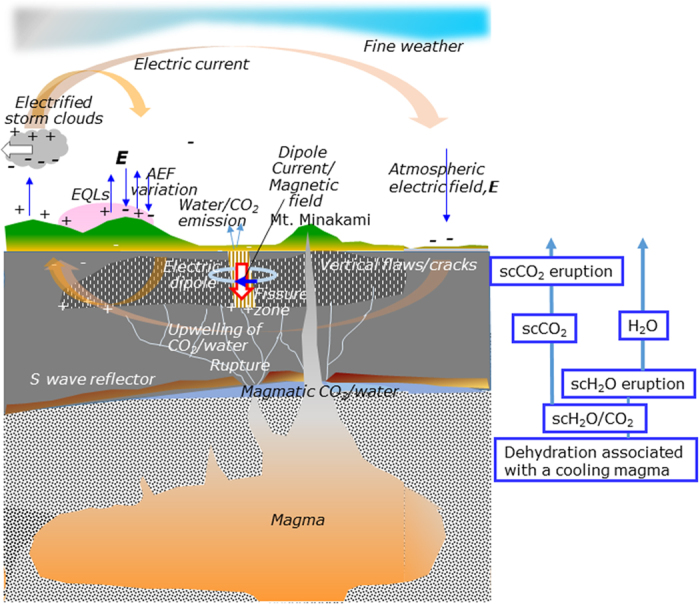
Causal mechanisms of seismo-EM phenomena during the 1965–1967 Matsushiro earthquake swarm. The flow diagram to the right indicates the phase change and the migration of the fluids, water/CO_2_, from deep below the surface. Orange-colored arrows indicate the atmospheric electrical current driven by coupling with thunderstorm and seismic-dipole activities which cause EQLs and AEF variations. The open red-coloured arrow in the fault zone indicates a seismic-dipole current inducing geomagnetic variation.
