# Supplementary material for: Causal mechanisms of seismo-EM phenomena during the 1965–1967 Matsushiro earthquake swarm
Source: Sci Rep. 2017 Mar 21;7:44774. doi: 10.1038/srep44774 (PMC5359576; doi:10.1038/srep44774)
Supplement: Supplementary Information [file srep44774-s1.pdf]

Supplementary information

Causal mechanisms of seismo-EM phenomena during the 1965-1967 Matsushiro earthquake swarm

Yuji Enomoto[1], Tsuneaki Yamabe[1] and Nobuo Okumura[2]

[1]Shinshu University, Ueda Campus, 3-15-1 Tokida, Ueda, Nagano 386-8567 Japan

[2]Genesis Research Institute, Inc., 4-1-35 Noritake-shinmachi, Nishi-ku, Nagoya, Aichi 451-0051 Japan

Additional data of the fracture tests

In order to ensure the crack could initiate from the upper side to allow CO_2 gas to flow, as shown in Supplementary Fig.1a, an initial flaw of 8 mm long × 0.4mm width (c.f. insert in the bottom left) was introduced on the rock surface just beneath the open slit of the chisel using a small-sized grinder blade, and the rock sample was lightly pressured with a metal plate and four clamps at the corners. In some tests, a reference electrode was set on the base rock to detect any noise signals excluding the electrified gas current.

All data of the electric currents included in Fig.4b in the article are shown in Supplementary Fig 1b-I, where green lines are the electrified gas current, orange ones are the signal from the reference electrode, and gray ones are the signal from the vibration sensor. In some cases, the spike-like signals in phase opposite to the electrified gas current appeared at the moment of final rupture (c.f. Supplementary Fig.1 b, c and d). Since the spike-like signals of the reference electrode are synchronized with the electrified gas current signals, they might be attributed to displacement currents due to the sudden electric field generation between the charge-separated mating fracture surfaces.

Some fracture tests using less-quartz bearing gabbro were also conducted for comparison with quartz bearing diorite. As shown in Supplementary Fig.1j, the electrified gas current could be measured, of which the intensity fell on the relationship of Eq. (1) in the article.

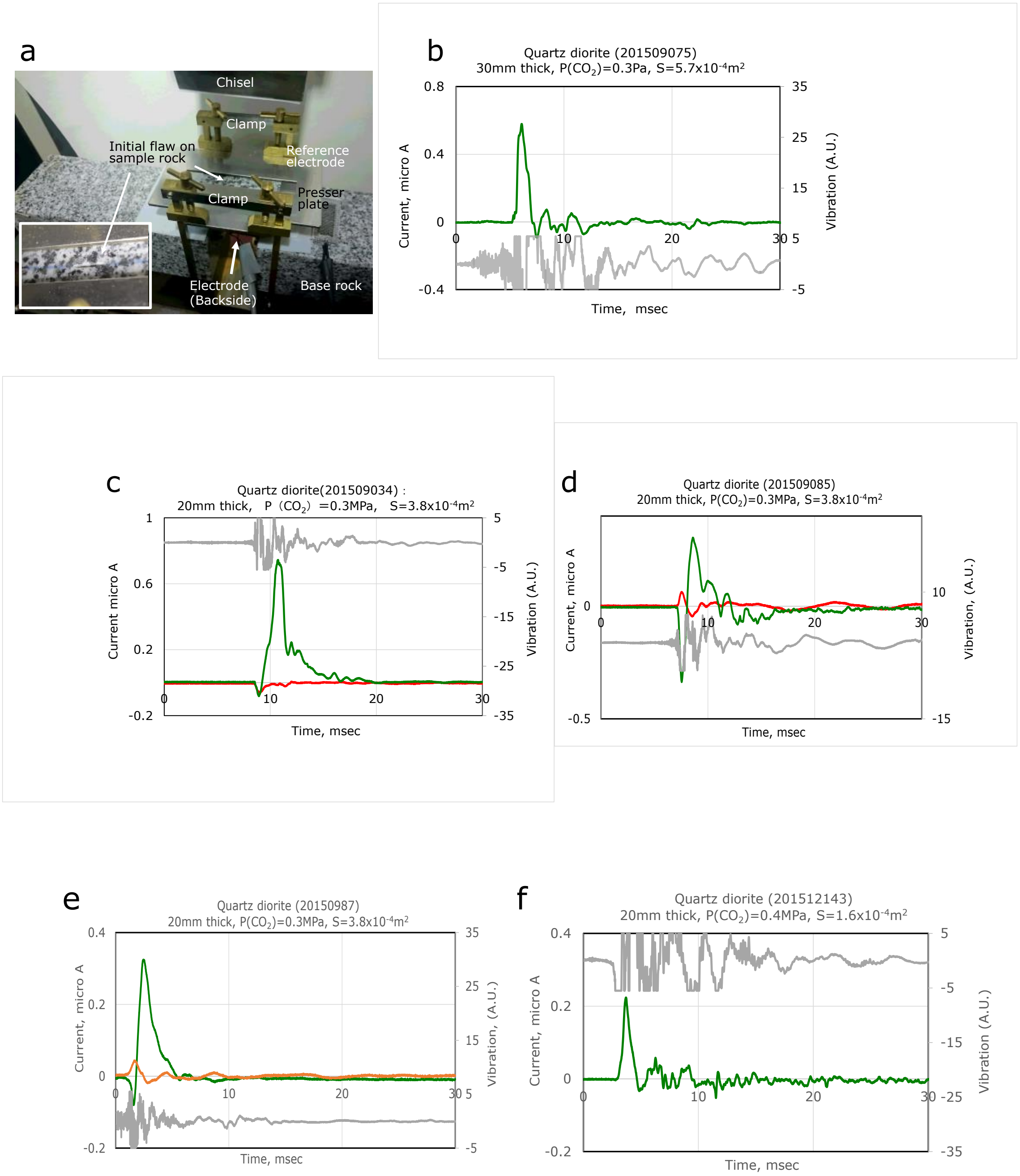
a
Chisel
Clamp
Initial flaw on sample rock
Reference electrode
Presser plate
Clamp
Electrode (Backside)
Base rock
b
Quartz diorite (201509075)
30mm thick, P(CO2)=0.3Pa, S=5.7x10-4m2
Current, micro A
Vibration (A.U.)
Time, msec
c
Quartz diorite(201509034) :
20mm thick, P (CO2) =0.3MPa, S=3.8x10-4m2
Current micro A
Vibration (A.U.)
Time, msec
d
Quartz diorite (201509085)
20mm thick, P(CO2)=0.3MPa, S=3.8x10-4m2
Current, micro A
Vibration, (A.U.)
Time, msec
e
Quartz diorite (20150987)
20mm thick, P(CO2)=0.3MPa, S=3.8x10-4m2
Current, micro A
Vibration, (A.U.)
Time, msec
f
Quartz diorite (201512143)
20mm thick, P(CO2)=0.4MPa, S=1.6x10-4m2
Current, micro A
Vibration (A.U.)
Time, msec

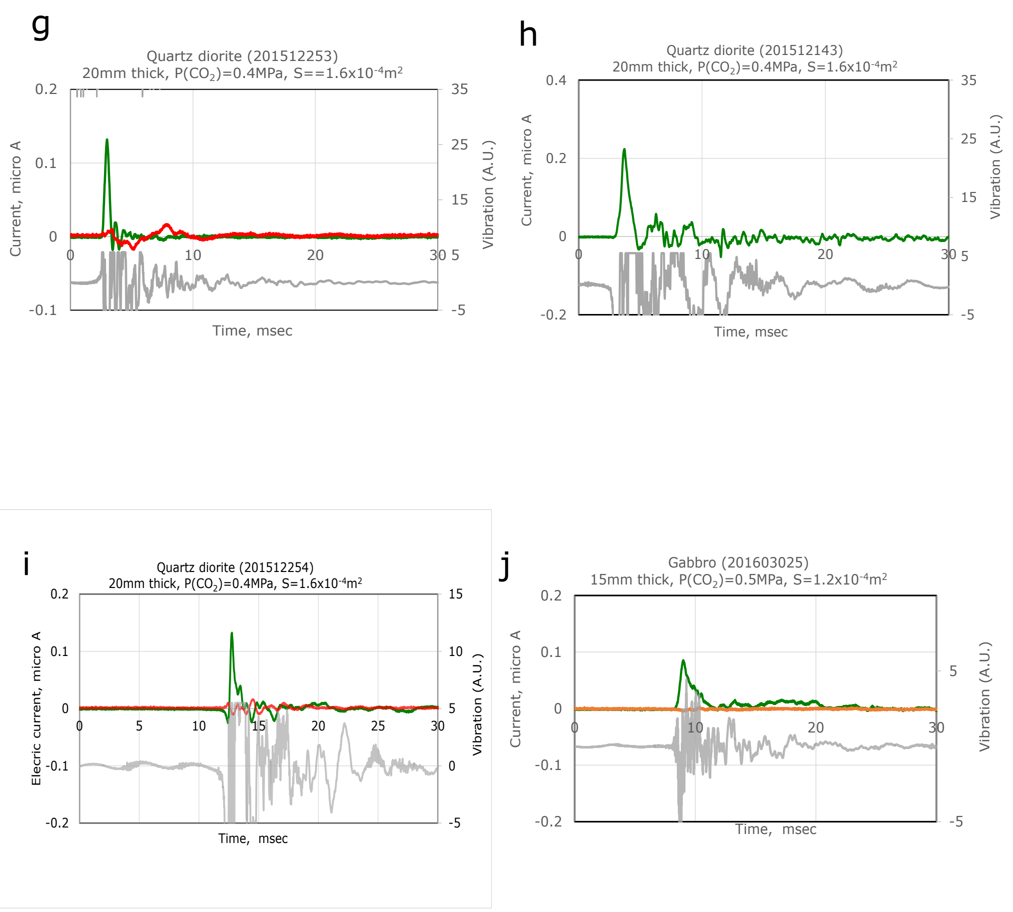

g
Quartz diorite (201512253)
20mm thick, P(CO2)=0.4MPa, S==1.6x10-4m2
Current, micro A
Vibration (A.U.)
Time, msec
h
Quartz diorite (201512143)
20mm thick, P(CO2)=0.4MPa, S=1.6x10-4m2
Current, micro A
Vibration (A.U.)
Time, msec
i
Quartz diorite (201512254)
20mm thick, P(CO2)=0.4MPa, S=1.6x10-4m2
Elecric current, micro A
Vibration (A.U.)
Time, msec
j
Gabbro (201603025)
15mm thick, P(CO2)=0.5MPa, S=1.2x10-4m2
Current, micro A
Vibration (A.U.)
Time, msec
